# Sustainable low-field cardiovascular magnetic resonance in changing healthcare systems

**DOI:** 10.1093/ehjci/jeab286

**Published:** 2022-02-14

**Authors:** Cathy Qin, Sanjana Murali, Elsa Lee, Vaishnavi Supramaniam, Derek J Hausenloy, Johnes Obungoloch, Joanna Brecher, Rongyu Lin, Hao Ding, Theophilus N Akudjedu, Udunna C Anazodo, Naranamangalam R Jagannathan, Ntobeko A B Ntusi, Orlando P Simonetti, Adrienne E Campbell-Washburn, Thoralf Niendorf, Regina Mammen, Sola Adeleke

**Affiliations:** 1 Department of Imaging, Imperial College Healthcare NHS Trust, London, UK; 2 School of Medicine, Faculty of Medicine, Imperial College London, London, UK; 3 School of Medicine, King’s College London, London, UK; 4 Division of Medicine, University College London, London, UK; 5 Cardiovascular & Metabolic Disorders Program, Duke-National University of Singapore Medical School, Singapore, Singapore; 6 National Heart Research Institute Singapore, National Heart Centre Singapore, Singapore, Singapore; 7 Yong Loo Lin School of Medicine, National University Singapore, Singapore, Singapore; 8 Hatter Cardiovascular Institue, UCL Institute of Cardiovascular Sciences, University College London, London, UK; 9 Cardiovascular Research Center, College of Medical and Health Sciences, Asia University, Taichung, Taiwan; 10 Department of Biomedical Engineering, Mbarara University of Science and Technology, Mbarara, Uganda; 11 Medical Education, King George Hospital, Ilford, UK; 12 School of Medicine, University College London, London, UK; 13 Institute of Medical Imaging and Visualisation, Faculty of Health and Social Science, Bournemouth University, Poole, UK; 14 Lawson Health Research Institute, London, Ontario, Canada; 15 Department of Electrical Engineering, Indian Institute of Technology, Chennai, India; 16 Department of Radiology, Sri Ramachandra University Medical College, Chennai, India; 17 Department of Radiology, Chettinad Hospital and Research Institute, Kelambakkam, India; 18 Department of Medicine, University of Cape Town and Groote Schuur Hospital, Cape Town, Western Cape, South Africa; 19 Division of Cardiovascular Medicine, Department of Internal Medicine, College of Medicine, The Ohio State University, Columbus, OH, USA; 20 Department of Radiology, College of Medicine, The Ohio State University, Columbus, OH, USA; 21 Cardiovascular Branch, Division of Intramural Research, National Heart, Lung and Blood Institute, National Institutes of Health, Bethesda, MD, USA; 22 Berlin Ultrahigh Field Facility (B.U.F.F.), Max-Delbrück Centre for Molecular Medicine in the Helmholtz Association, Berlin, Germany; 23 Department of Cardiology, The Essex Cardiothoracic Centre, Basildon, UK; 24 School of Cancer & Pharmaceutical Sciences, King’s College London, Queen Square, London WC1N 3BG, UK; 25 High Dimensional Neurology, Department of Brain Repair and Rehabilitation, UCL Queen Square Institute of Neurology, University College London, London, UK

**Keywords:** MRI, Low field, Sustainable, Global Health, Technology

## Abstract

Cardiovascular disease continues to be a major burden facing healthcare systems worldwide. In the developed world, cardiovascular magnetic resonance (CMR) is a well-established non-invasive imaging modality in the diagnosis of cardiovascular disease. However, there is significant global inequality in availability and access to CMR due to its high cost, technical demands as well as existing disparities in healthcare and technical infrastructures across high-income and low-income countries. Recent renewed interest in low-field CMR has been spurred by the clinical need to provide sustainable imaging technology capable of yielding diagnosticquality images whilst also being tailored to the local populations and healthcare ecosystems. This review aims to evaluate the technical, practical and cost considerations of low field CMR whilst also exploring the key barriers to implementing sustainable MRI in both the developing and developed world.

## Is there a need for low-field cardiac magnetic resonance imaging?

Cardiovascular disease (CVD) remains the leading cause of mortality worldwide, accounting for almost one-third of deaths and 330 million years of life lost in 2017 globally,^1^ with nearly 80% of CVD deaths occurring in low- to mid-income countries (LMICs).^2^ An essential contributor to CVD mortality and healthcare burden in LMICs is the limited accessibility to diagnostic imaging and screening^3^ as well as sufficiently trained human resources in image acquisition and interpretation. LMICs are expected to experience the steepest epidemiological transition from infectious disease to non-communicable disease (NCD) in the next two decades. Yet, these countries are also the least equipped with healthcare infrastructure.^4^ It is imperative to develop sustainable healthcare technology and policies to cope with the rising burden of NCD to meet the 2030 United Nations’ Sustainable Development Goals (SDG).^5^

Cardiovascular magnetic resonance (CMR) is well established as an essential imaging modality in evaluating cardiac anatomy and function.^6^^,^^7^ CMR is non-invasive, non-ionizing, and possesses multi-contrast and multi-parametric capabilities, allowing it to be an ideal modality in evaluating a diverse range of cardiac diseases from cardiomyopathies and coronary artery disease (CAD)^8^^,^^9^ to acute myopericarditis secondary to COVID-19.^10^^,^^11^ Early advances in CMR took advantage of magnetic field strengths limited to between 0.05 and 0.35 T. Still, image quality was hampered by the lack of high-performance gradient systems and sophisticated imaging techniques or pulse sequences.^12^ The last three decades have seen an accelerating trend towards increasing magnetic field strength with superior hardware and software to reduce examination times and environmental impact.^12^^,^^13^ In general, high-field (HF) magnetic resonance imaging (MRI) (1.5–7 T) accounts for the most significant global market share in 2020 (MRI systems market worth).^14^ Increasingly, ultrahigh field (UHF, *B*_0_ >7 T) systems are expected to experience a reasonable rate of growth by market value over the next few years as they begin to enter the clinical imaging domain.^15–17^

**Figure 1:**
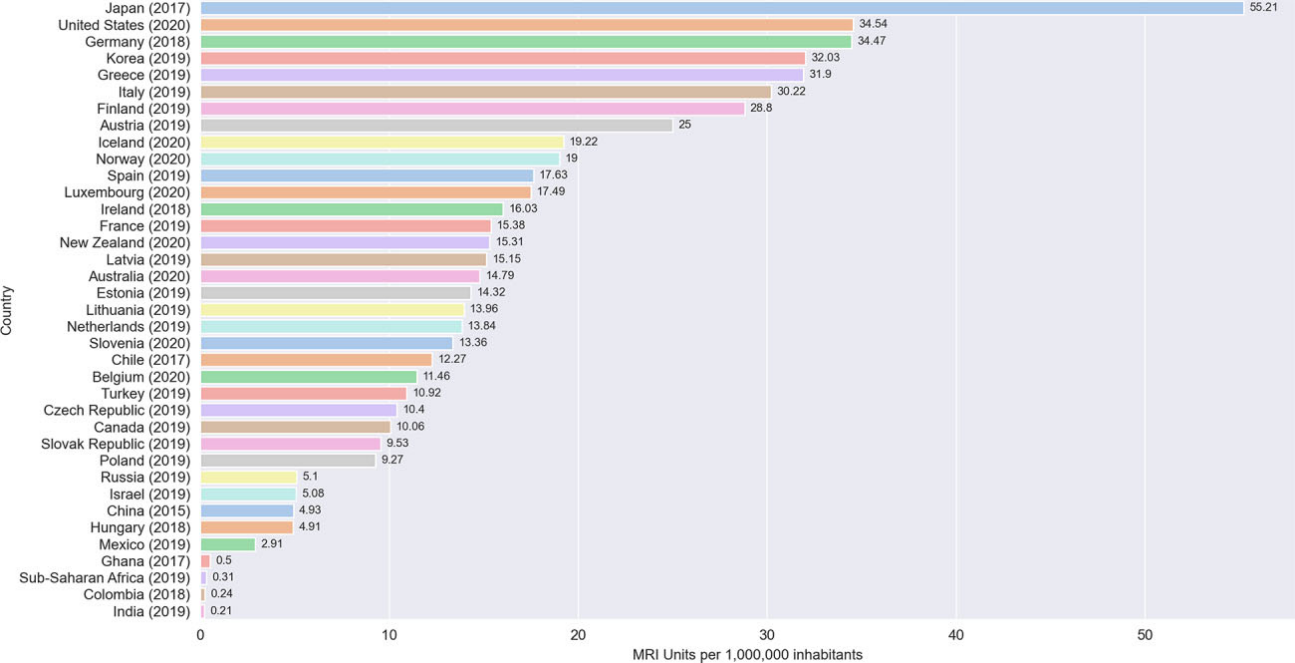
Bar chart displaying the MRI units per million population, as recorded by the Organization for Economic Co-operation and Development (OECD). The data are organized by density, with Japan having the highest density at 55.21 units per million population.

**Figure 2:**
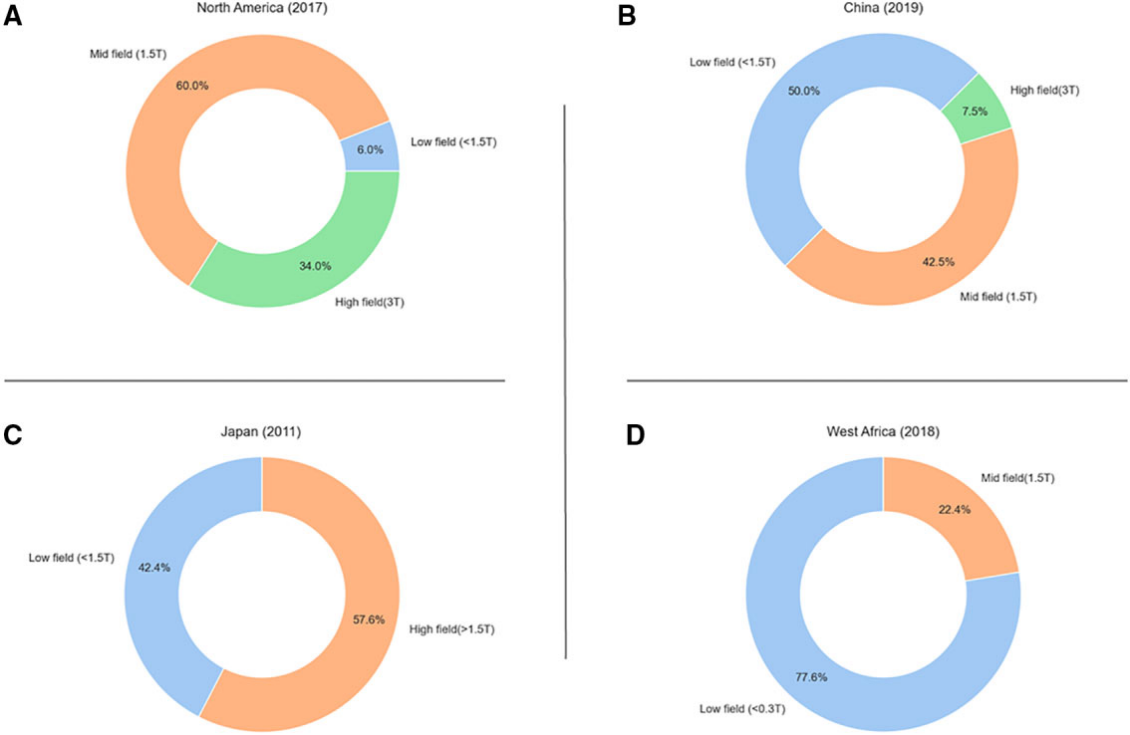
The proportion of low-, mid-, and high-field MRI scanners of selected countries. *From left to right*: (*A*) the distribution in North America as of 2017, (*B*) the distribution in China as of 2019, (*C*) the distribution in Japan as of 2011, and (*D)* the distribution in West Africa as of 2018.

However, there is an extreme global disparity in MRI availability and accessibility, meaning these state-of-the-art units remain a cost-intensive luxury. As seen in *Figure 1*, Europe and North America have a high density of MRI units at 22.2 per million population (pmp).^17^ In stark contrast, Sub-Saharan Africa (SSA) has an average of 0.3 MRI units pmp,^17^ with 11 countries with populations ranging from 0.7 million to 67.5 million having no scanners at all.^18^*Figure 2D* displays West African survey results, which showed over 75% of available scanners in this region were low-field (LF, *B*_0_ <1.5T) systems, with the remaining scanners being of 1.5 T in strength. As of 2018, there were no 3 T scanners in this region.^19^ On average, Asia has a wide distribution of scanner density; at 45.94, Japan has the highest number of MRI units pmp^20^ with a relatively high proportion of low-mid field scanners.^21^*Figure 2B* shows that similarly, in China, over 50% of scanners are of the LF range (<1.5 T) compared to only 6% in Europe and North America.^22^^,^^23^ On the contrary, India has <1 unit pmp to service its dense population of 1.32 billion.^18^ Despite the existence of MRI units for other body systems in LMICs, CMR-capable scan protocols, RF coils, and set-ups are in short supply. Only eight countries provide CMR services on the African continent, with most scanners concentrated in South Africa and limited to the private sector and academic centres.^24^ In contrast, in the UK, 112 centres offer CMR services.^25^

CMR scanners require highly trained personnel and a significant budget to operate and maintain. A considerable proportion of the cost of an MRI unit lies in the acquisition of the superconducting magnet, which is valued at up to 1 million euros (1.2 million USD) per tesla^13^ end-user, making it one of the most expensive pieces of machinery in a hospital. Furthermore, the plethora of functionalities makes a CMR test time-consuming, with a typical scan taking 60 min.^26^ This severely limits accessibility in resource-poor countries, further compounded by limitations in geographical access, unreliable power supply, and deficits in education and training.^18^

The last decade has seen rapid developments in sustainable MRI technology, focusing on reducing costs without compromising performance. Examples include lighter, cryogen-free magnet design, improved gradient coil configuration, and performance and sophisticated image acquisition and reconstruction software. Such developments can regain much of the spatial resolution lost at LF strengths. Recent work, spearheaded by Campbell-Washburn *et al*., has also demonstrated the feasibility of employing widely used CMR sequences such as 2D CINE imaging of the heart using high blood-myocardium contrast imaging techniques on LF systems. This helps achieve diagnostic-quality cardiac images in a reasonable timeframe.^27–31^

Furthermore, LF CMR may prove economical for evolving LMICs healthcare ecosystems. Simplifying MRI hardware and software are key to achieving sustainable and affordable scanning units.^32^ By using permanent, non-cryogenic magnets with a wide-bore or open configuration, LF units have the potential to significantly reduce costs, whilst also increasing patient comfort and improving access.^32–34^ With the ever-increasing global demand for diagnostic imaging, coupled with growing concerns about climate change and helium shortages, it is critical for the MRI community to invest in sustainable technology to meet this demand, whilst minimizing environmental impact. Capitalizing on LF MRI technologies is arguably a viable and effective solution to address these issues going forward.^27–31^

This review explores the various opportunities and challenges for implementing sustainable LF CMR in different healthcare ecosystems by evaluating the technical, practical, and cost considerations. We postulate a future where LF CMR is potentially a viable, non-inferior alternative to standard field CMR, which is suitable and fit-for-purpose, especially in rapidly changing ecosystems such as LMICs and rural/deprived regions of high-income countries (HICs) where access to diagnostic imaging is limited.

## What is the need for improved imaging services in LMICS?

Eighty per cent^35^ of the global burden of CVD is in LMICs, with an increasing shift towards non-communicable causes of CVD-related diseases.^36^^,^^37^ Similar to the HICs, ischaemic heart disease (IHD) and stroke are now the leading causes of CVD-related mortality in LMICs,^38^ whilst the non-ischaemic causes of heart failure and premature cardiovascular mortality persist, e.g. hypertension, rheumatic heart disease (RHD), Chagas disease, Human Immunodeficiency Virus (HIV), and other infectious causes of endomyocardial fibrosis, effectively placing a ‘double burden’ on the economy and healthcare infrastructures.^3^^,^^39–41^ LMIC’s mortality rates are considerably higher than HICs despite having younger patient cohorts and lower comorbidities.^40^^,^^42^ CVD disproportionately affect the working-age population in LMICs and precipitate substantial national economic loss as shown in macroeconomic studies; in SSA, $9 billion or 7% GDP loss secondary to CVD is reported in 2001.^43^ Between 2013 and 2030, this is projected to be $2.4 trillion in India and $8.8 trillion in China.^44^ Therefore, early detection of CVD through diagnostic imaging is essential for initiating primary/secondary prevention or early therapeutic intervention.^45^

## How can challenges in access to CMR be addressed in diverse economies?

### CMR in LMICs

Financial restrictions and resource availability heavily compromise access to sophisticated imaging in LMICs. Inadequate healthcare spending exacerbates disease burdens in LMICs; as shown in a systemic review, annual healthcare costs of CVD greatly exceed health expenditure per capita in most LMICs.^46^ In certain LMICs, such as in SSA, there is a disproportionately small allocation of the total global health budget to NCDs, including CVD, despite its increasing weight.^47^^,^^48^ The challenge is made more difficult by a lack of imaging facilities in LMICs with significant inequitable MRI scanner distribution. Within countries, most MR scanners are concentrated in major cities^49–52^ and are mostly privately owned.^50^^,^^53^ This is especially striking in South Africa, where the private sector possesses >90% of MRI scanners and yet caters to only ∼16% of the population.^54^ In 2012, 84 MRI scanners served public health insurance patients in Brazil, and 1263 MRI scanners were at the country’s private practices.^55^

Scarce data exist on CMR availability and utilization in LMICs. Unpublished data by Anazodo *et al*.’s CAMERA survey on MRI indications across 91 sites with MRI facilities on the African continent found only 5 sites reported cardiovascular indications as one of the common indications for MRI. A recent survey on the infrastructural gaps in diagnostic imaging for congenital heart disease across 34 locations in 17 different LMICs (including the Americas, Asia, and Eastern Europe) found that only 54% provided CMR services.^56^ A 2018 study summarizing the number of attendees at the Society of Cardiac Magnetic Resonance’s annual scientific meetings over the last 20 years found that unsurprisingly, a much smaller proportion of attendees came from LMICs compared to HICs. For instance, over 6 years, 14.7 attendees came from Africa and the Middle East, 19.4 came from Central and South America, and 641.0 attendees came from the USA and Canada. This data indirectly highlight the sheer lack of availability and underutilization of CMR in LMICs, and when available, are mainly used for academic research.^57^ The high acquisition and ongoing maintenance costs of CMR pose significant obstacles to adoption, which is further compounded by healthcare worker shortages, limited technical expertise, such as engineers and medical physicists to service and repair such devices.

Furthermore, relevant imaging specialists, such as radiologists, are frequently not consulted in the purchasing process of imaging technology that must be appropriate and tailored to the local needs.^58^ This partly explains the high failure rate of donated equipment in LMICs.^59^^,^^60^ Device failure is a common issue as repair works are often carried out by specialist companies covering a large geographical area, which can incur substantial delays. Additional factors, such as long scan duration, inadequate transport infrastructure, insufficient emergency medical services, network connectivity failure, and environmental constraints, may exacerbate the matter. The long scanning duration in CMR also contributes to underuse in LMICs.^61^^,^^62^

A sufficiently trained workforce is essential for running any radiology service. There is a lack of formal radiology training programmes in LMICs^36^; a survey of 13 African countries found that 62% of countries surveyed offered <5 radiology residencies, whilst only 2 countries offered subspecialty training.^63^ A Latin American survey found training programmes in 7 out of 17 countries offered provided subspecialty training.^63^^,^^64^ A recent survey of paediatric cardiologists in Brazil found that 79% of respondents had access to CMR, of whom 52% rarely or never use it (40% response rate). The main barrier to its more frequent use was identified as a shortage of qualified professionals (55%).^65^ A sustainable MRI service demands more than just the initial set-up cost. A robust ecosystem of healthcare workers, technical support, regulatory and safety frameworks, organizational planning, and well-designed national policies for upscaling and delivering imaging services is required.^66^

### CMR in HICs

Significant disparities exist in how CMR and imaging services are utilized in HIC.^61^ One manifestation is in accessibility between cities vs. rural regions.^62^^,^^67–71^ In 2018, over 39 000 CMR scans were performed in London, UK, compared to 17 000 cases in the Midlands (a relatively rural region in the UK) despite having a similar number of scanners, 2.9 vs. 2.8 scanners PMP, respectively.^62^ The geographical expanse of rural areas compared to urban ones, consequently affecting patient access to CMR, may contribute to the disparity in the utilization of CMR, making a potential argument for increasing the density of CMR scanners in rural areas. In addition, it was noted that the mean outpatient waiting time for a CMR scan in London was 28 vs. 40.7 days for the Midlands.^58^ Local staffing and the expertise needed to report scans may be a reason for the disparity. This can be improved by pooling resources, offering support and proctorship for low-volume centres as encouraged by the national imaging board of the UK, the British Society of Cardiac MRI, outlined in their CMR imaging standards.^62^^,^^72^^,^^73^

Beyond a geographical divide, recent data from the USA has shown that there are imaging inequalities even in racial and ethnic minorities and in those from lower socio-economic groups.^67^ In addition, there are differences in healthcare offered to women with CVD and consequently in their clinical outcomes. This is partly due to gender differences in clinical presentation, pathophysiology, and diagnoses, e.g. in conditions, such as peripartum cardiomyopathy, MINOCA, Takotsubo cardiomyopathy, cardiac dysfunction related to chemotherapy, and systemic sclerosis.^65^^,^^67^ CMR has a unique safety advantage in diagnostic imaging in women due to its lack of ionizing radiation, whilst also offering early and accurate detection of these conditions. Recognizing the role CMR has to play, the Society of Cardiovascular Magnetic Resonance has released guidance, education, and information for the use of CMR in women with CVDs in a drive to address this gender disparity.^71^^,^^74–76^

## How can LF CMR be implemented?

The development of newer clinical grade, LF MRI scanners is a field of active research, populated by developments over the last few years. Although LF CMR does have a number of advantages, highlighted in *Table 1*, this section will describe the technical challenges that need to be surmounted for this technology to be available to users across various economic divides.

**Table 1:** Comparison of low-field and high-field MRI

	Low field (<1.5 T)	High field (3T<)
Cost	Lower initial purchase price (€30 000–€80 000 for 0.2 T) and lower operating costs^77^	High purchase cost (€400 000+) and higher operating costs^77^
SAR	Lower SAR meaning less energy deposited in tissue per radiofrequency pulse, making it safer for vulnerable individuals^78^	Higher SAR, meaning more energy deposited in tissue, leading to faster heating of tissue^78^
ECG gating	Less MHD interference allowing for distortion-free ECG trace^79^	High levels of MHD interference which impede MRI synchronization^79^
SNR	Lower SNR, which predisposes to reduced image quality^13^	Higher SNR, which leads to more accurate images with higher resolution^13^
Acoustics	Lower acoustic noise which makes it safer for operating staff and more comfortable for patients^80^	Higher level of acoustic noise^80^
Scan times	Longer acquisition time^13^	Shorter acquisition time^13^

CMR is a technically demanding imaging modality. Rapid imaging is critical to compensate for respiratory and cardiac motion. Typically, CMR sequences use high-performance gradients (high slew rate and amplitude) to achieve rapid imaging. Parallel imaging is routinely deployed to accelerate acquisitions. Due to proximity to the lungs, good magnetic field homogeneity is essential to limit susceptibility artefacts, especially banding and off-resonance artefacts in bSSFP-based 2D CINE acquisitions used for cardiac chamber quantification.

### Sensitivity and signal-to-noise considerations

An important factor that governs image quality in MRI is the Signal to Noise Ratio (SNR). SNR is the ratio of the MRI signal relative to the standard deviation of the background noise. Even though SNR scales supra-linearly with magnetic field strength [SNR ∼ *B*_0_ (1.65)],^81^ this gain is disproportionately small due to several factors such as increases in receiver bandwidth for the management of the enhanced fat-water chemical shift, T_1_ relaxation time prolongation, T_2_ and T_2_* relaxation time shortening, radio frequency (RF) attenuation, RF power deposition constraints and tissue conductivity at increasing magnetic field strengths, which counteract the increase in SNR. SNR can be optimized by improving RF coil design/geometry, leveraging modern image acquisition and reconstruction techniques, and increasingly with deep learning (DL) methods.^12^^,^^13^^,^^33^^,^^82^^,^^83^ These approaches are more cost-effective than investments into stronger but disproportionately expensive magnets, a significant cost-driver.

Image artefacts impede image quality. The SNR gain can be translated into enhanced image resolution and image granularity at higher field strengths. However, this makes image quality at higher fields more prone to bulk movement and physiological motion, including cardiorespiratory motion, pulsation, and beat-to-beat variations in blood flow. Furthermore, the higher spatial resolution demands longer scan durations, which can exacerbate movement artefacts. These can severely degrade image quality due to motion-induced ‘blurring’, ‘ghosting’, and ‘misregistration’, which may compromise image interpretation.^84^ SNR constraints at low magnetic fields can be offset by relaxing spatial resolution. This approach goes along with reducing the propensity for motion artefacts in LF MRI.

### RF power deposition considerations

At LF strengths, the specific absorption rate (SAR), which describes the amount of RF energy deposition in tissues, is significantly lower than at high magnetic field strengths. This allows for increased flexibility in adapting image protocols to boost SNR without breaching the SAR limits^85^ and thus, critical heating of tissue.^12^^,^^33^ This is especially useful in cardiac imaging, which employs SAR intense black blood imaging techniques for probing cardiac morphology or oedema imaging and tissue characterization.^86^ Low SAR also facilitates faster acquisitions and permits the utilization of higher flip angle CINE acquisitions, which can improve blood-myocardium contrast at lower fields for the benefit of enhanced cardiac chamber quantification and function assessment.^27^

### Magnet configurations

Most MRI systems employ superconducting solenoid magnets, which generate high magnetic field strength; these, however, require a regular supply of liquid helium, which is costly and non-renewable. Besides the magnet weight (up to 6000 kg for 1.5 T scanners), infrastructural demands such as stray field shielding requirements and a helium quench pipe installation increase magnetic footprint and limit portability.^32^^,^^34^^,^^87^ Modern cryocoolers employing Gifford–Mahon pulse tubes use direct conduction cooling, which allows for a dry or nearly dry system, reducing operational costs; the drawbacks are the need for regular maintenance and potential field disruption by mechanical vibrations.^34^ Another key strategy to reduce MRI cost and footprint includes reducing the bore diameter and configuring RF coils only around the organ system under investigation^88^; for instance, Panther *et al*.’s^89^ design of a head-only, conduction-cooled, 0.5 T scanner weighing just over 1100 kg.

Replacing the superconducting magnet with permanent magnets is an alternative. Permanent magnets have minimal energy requirements and absolve the need for a cooling system.^88^ Though traditional permanent magnet array set-ups are inherently heavy to maintain field homogeneity, recent work on Halbach arrays in neuroimaging has been shown to significantly reduce weight and lower costs.^32^^,^^90^^,^^91^ These strategies may find use in cardiac imaging, though optimization of gradient performance is essential to sustain the high demands of cine CMR imaging.^90–92^ It has also been proven that point-of-care MRI scanners that utilize superconductive magnets are feasible clinical imaging solutions.

**Figure 3:**
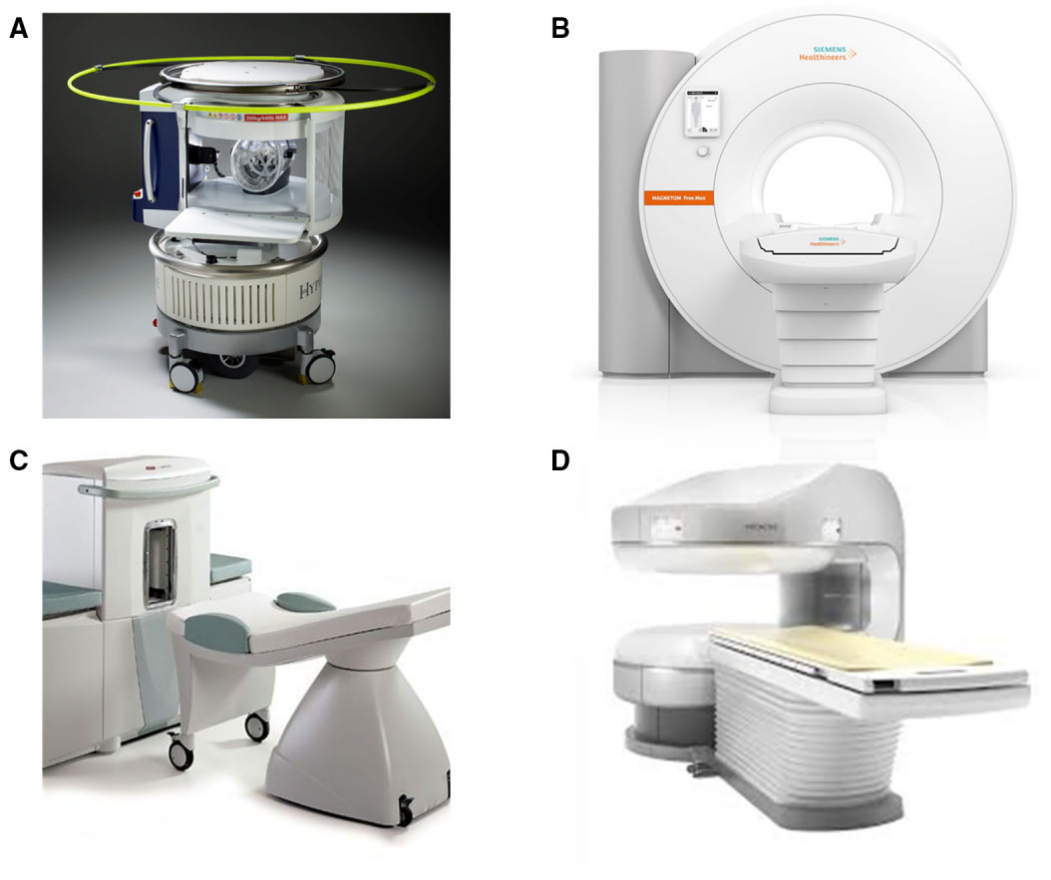
*From left to right, top row –* (*A*) hyperfine 0.064 T *(image courtesy of Hyperfine)*, designed to deliver point of care brain imaging. In this instance, the scanner would need to be optimized for use in a cardiac setting. (*B*) The Siemens Free.Max 0.55 T scanner *(image courtesy of Siemens).* These illustrate the size of the 80 cm bore, which is optimized to provide more comfort to patients when undergoing MRI treatment, or for those who cannot tolerate typical bore sizes. *Bottom row—*(*C*) Esaote C-Scan from Esaote, which scans at 0.2 T and is optimized for musculoskeletal injuries.^94^ (*D*) Aperto Lucent Plus 0.4 T from FUJIFILM, which has the advantage of being an open bore scanner for more comfort for patients.^95^

An example of this is the Hyperfine 0.064 T, which uses two horizontally orientated permanent magnets to form the poles for the system.^93^*Figure 3*displays the current model for the Hyperfine, which needs optimizing and remodelling for cardiac imaging. These lightweight, low-cost permanent magnet designs can potentially be portable^96^ and may find use in CMR if bore size is increased.

### Open MR scanners

Patients with claustrophobia, high body mass index, and paediatric populations may benefit from open configuration LF scanners. This may also facilitate patient monitoring and interventional procedures.^97^ Additionally, open scanners may allow for upright in-scanner exercise CMR, preventing heart rate recovery between exercise cessation and image acquisition. However, this technique is limited by movement artefacts and electrocardiogram (ECG) interference.^98^^,^^99^ Very few studies have evaluated open CMR. Klein *et al*.^97^ found an open 0.35 T system offered adequate image for functional CMR but required higher field strengths for perfusion and viability assessments in 11 patients. Studies on LF (1 T) open CMR generally report reduced SNR and CNR compared to standard 1.5 T closed bore scanners but comparable subjective image quality.^100–103^ Recent technology has been expanding to improve overall patient comfort. The Siemens Free.Max is a 0.55 T system with a conventional, superconducting solenoid design that incorporates a larger bore size of 80 cm, as seen in *Figure 3*. This offers the potential that lower field systems can have cost-effective increases in bore size.^104^

### Gradient coil performance and acoustic noise

Many routine CMR imaging techniques require rapid gradient switching, yet most commercially available lower field systems (permanent and electromagnets) lack sufficient gradient performance.^12^ Current research on LF CMR has leveraged the superior gradient performance of standard 1.5 T superconducting magnets, modified to operate at LF. The minimum gradient performance needed to generate diagnostically useful cardiac images in a reasonable timeframe remains unknown, and indeed, more research is warranted to establish this.

Acoustic noise induced by the pulsed gradients is reduced at LF vs. HF. Doubling the magnetic field amplifies the acoustic noise level generated by the gradient coils by ∼6 dB (logarithmic scale).^105^ A closer examination of the gradient noise showed acoustic noise levels of 77 dB at 0.5 T. For the 1.5 T counterpart, acoustic noise levels of 98 dB were observed.^105–107^ A team spearheaded by L.L. Wald has recently established a portable LF prototype scanner (weight: 122 kg, *B*_0_ = 80 mT). This configuration employs a built-in magnetic field gradient.^96^ This approach reduces the reliance on high-power gradient drivers and lowers acoustic noise levels due to the elimination of a readout gradient coil. For this set-up, A-weighted peak (75.4 dB) and average sound pressure levels (69.3 dB) were reported for rapid acquisition and relaxation enhancement (RARE, i.e. fast spin-echo) imaging.^96^^,^^108^^,^^109^ Combining the inherent gradient approach with sweep imaging with silent MR techniques promises to reduce further if not eliminate acoustic noise.^110^ To summarize, reducing acoustic noise exposure at LF improves patient comfort and makes it conceptually appealing to pursue CMR in neonates and young infants without general anaesthesia.^111^

### Magnetic susceptibility

Susceptibility is loosely defined as the magnitude of polarization in materials or tissue in the presence of an external magnetic field, which either augments or weakens the external field. Most biological tissues are weakly diamagnetic, whereas ferromagnetic materials, e.g. iron and steel alloys found in metallic foreign bodies and surgical implants, have very high magnetic susceptibility. Imaging in the presence of these materials induces local field inhomogeneities creating severe image artefacts.^112^ Susceptibility artefacts are significantly reduced at LFs,.^12^^,^^33^^,^^113^^,^^114^

### MR safety of implants and devices at LFs

At LF, the RF wavelength (*λ*) in myocardial tissue and blood is substantially prolonged (*λ* ∼153 cm for *B*_0_ = 0.55 T) compared to high magnetic fields (*λ* ∼55 cm for *B*_0_ = 1.5 T), which^115–117^ reduces the risk for metallic implant heating.^8^^,^^23^^,^^33^^,^^47^ Currently, only several cardiac implantable electronic devices (CIEDs) are designed to be MR-safe/conditional with data registries. However, the majority of CIEDs *in situ* worldwide have not received regulatory approval for MRI. A recent multi-centre study concluded that ‘there is no incremental risk of either clinical safety events or early changes to device or lead performance from 1.5 T MRI for patients with non-MR conditional pacemaker or defibrillator leads compared with those labelled MR-conditional, when approved protocols are followed’.^118^ This work suggests that CMR at 1.5 T can be performed safely on ‘legacy’ devices given sufficient on-site electrophysiology support and the use of standardized MRI protocols.^50^^,^^51^

Scarce data on using LF MRI with cardiac devices show a comparably favourable safety profile with minimal patient-reported side effects, reduced RF-heating, and no statistical change in device parameters. Additionally, images generated were of good quality with reduced susceptibility artefact.^33^^,^^52^^,^^53^

In conjunction with the similar safety profile at LF, this observation allows for timelier imaging and thus faster access to medical treatment for patients with cardiac implants. These findings and opportunities render LF-CMR an attractive platform for imaging-guided interventions as device heating is reduced ∼7.5-fold compared to 1.5 T.^113^

### Cardiac triggering and gating

**Figure 4:**
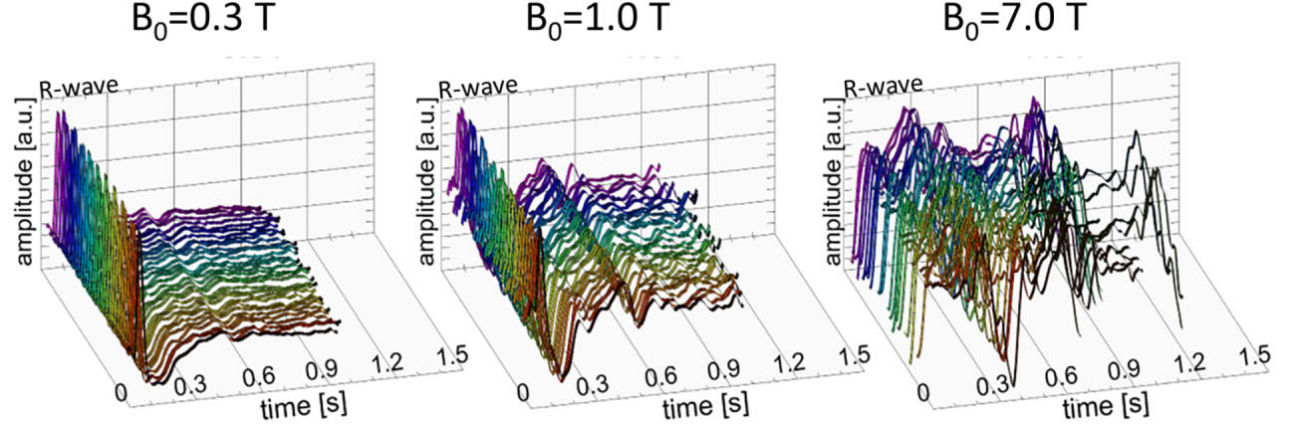
ECG traces obtained at *B*_0_ = 0.3 T, *B*_0_ = 1.0 T, and *B*_0_ = 7.0 T using three-lead vector ECG. ECG, an inherently electrical measurement, is prone to interferences with electromagnetic fields and magnetohydrodynamic (MHD) effects. The MHD effect scales with the magnetic flux density, flow orientation with respect to the magnetic field lines, and velocity of an electrical charge carrier such as blood. The MHD effect creates electric potential, which is superimposed onto the ECG potential. At *B*_0_ = 0.3 T, the ECG trace is mainly free of distortions. At *B*_0_ = 1.0 T, adverse signal elevation is found in the ECG for cardiac phases where typically the T-wave occurs. These artefacts are pronounced at *B*_0_ = 7.0 T. MHD induced artefacts in the ECG trace, and T-wave elevation might be misinterpreted as R waves resulting in erroneous triggering together with motion corrupted image quality. This issue is pronounced at higher magnetic fields. These artefacts render MHD effects detrimental for reliable synchronization of MRI or image registration with the cardiac cycle and constitute a practical impediment (Original Image from Ref.^79^).

Current routine clinical CMR is not a real-time imaging modality. MRI of a dynamic organ like the heart requires accurate synchronization of MR signal acquisition to the cardiac cycle. This is typically achieved using prospective ECG triggering or retrospective gating to acquire data segments over a series of cardiac cycles or R-R intervals. At increasing magnetic field strengths, the electromagnetic field and the magnetohydrodynamic (MHD) effect interfere with the ECG signal. This leads to misrecognition of the R wave (*Figure 4*), which severely disrupts cardiac gating.^79^^,^^119^ Whilst strategies such as acoustic triggering mitigate the MHD effect at HFs,^119^ MHD is substantially reduced at LFs (*Figure 4*), which may permit accurate 12-lead ECG monitoring of the patient during scanning and ECG triggering/gating.^120^

### Scanning times

At LF strengths, data averaging and consequently increasing scanning time may compensate for the SNR penalty; this may increase patient discomfort, bulk and physiological motion artefacts, and limit throughput.^121^ In practice, the loss of SNR is less than expected and depends on the imaging technique or protocol used. Scanning times can be minimized by utilizing receive RF coil arrays for parallel imaging and leveraging modern sequence/reconstruction strategies that focus on data sampling efficiency and compressed sensing reconstructions.^83^^,^^122–124^

## Can LF CMR generate diagnostic quality images?

**Figure 5:**
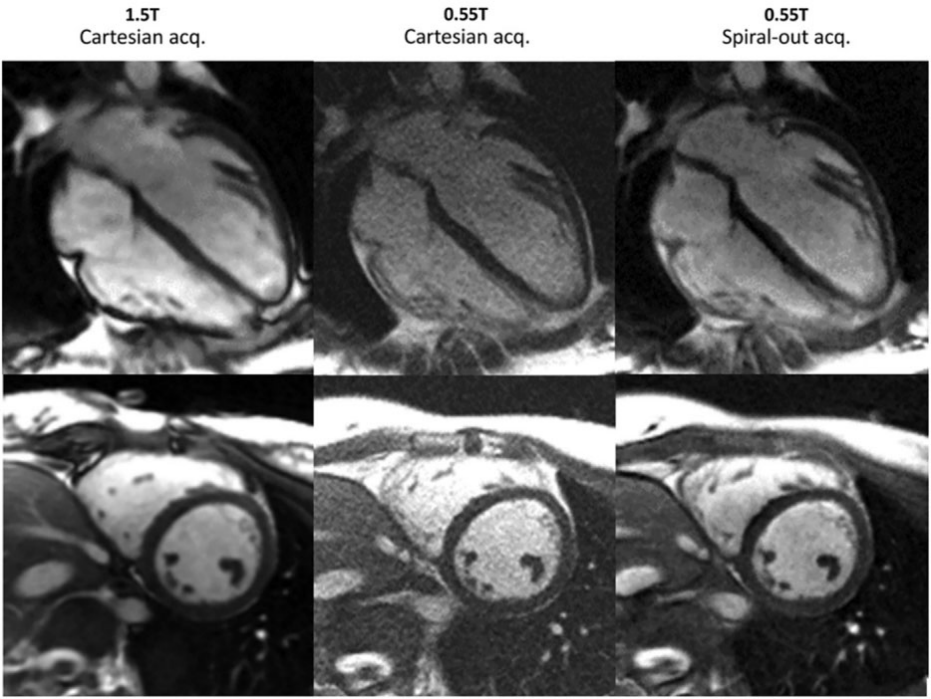
Example of boosting SNR using spiral-out acquisition to enhance sampling efficiency at low-field bSSFP CMR in a 23-year-old woman (Original Image from Ref.^113^).

A diagnostically useful image needs to be of sufficient resolution and quality to answer the clinical question in an acceptable timeframe. Recent publications have investigated the diagnostic capability of LF CMR that leverage high-performance gradients systems on superconducting magnets. As seen in *Figure 5*, Campbell-Washburn *et al*. modified a 1.5 T superconducting system to operate at 0.55 T while maintaining software and hardware capabilities, including 45 mT/m maximum gradient amplitude. Only a small subset of patients had CMR in this study, and 57% SNR was achieved in 11 patients.^113^ Restivo *et al*. used this system with spiral in-out bSFFP acquisitions to show that SNR of the myocardium at 0.55 T reached almost 70% of SNR at 1.5 T, though SNR of blood at 0.55 T reached just over half of that achieved at 1.5 T. However, total acquisition time did not increase, and the sequence was resistant to motion and flow artefact.^30^ Bandettini *et al*. acquired paired images using a 1.5 T CMR scanner and 0.55 T in 65 subjects (44 clinically referred) with matched image acquisition time. There were no significant differences in volumetric chamber assessments. There was also close agreement (kappa 0.99) in identifying regional wall motion abnormalities between the two field strengths. SNR of blood, myocardium, and relative CNR at 0.55 T reached ∼50% of that achieved at 1.5 T using a breath-held cine sequence. A free-breathing cine sequence with compressed sensing reconstruction was also demonstrated to improve image quality. There were good-to-excellent diagnostic confidence scores for 0.55 T images despite slightly higher mean scores at 1.5 T.^31^ More recently, the same group evaluated the performance of late gadolinium enhancement (LGE) at 0.55 T using bSSFP readout compared to gradient-echo readout at 1.5 T in 12 patients with myocardial infarction (MI). Both qualitative and quantitative measurements of MI were comparable across the two field strengths indicating the feasibility of evaluating myocardial viability at LF.^29^

**Figure 6:**
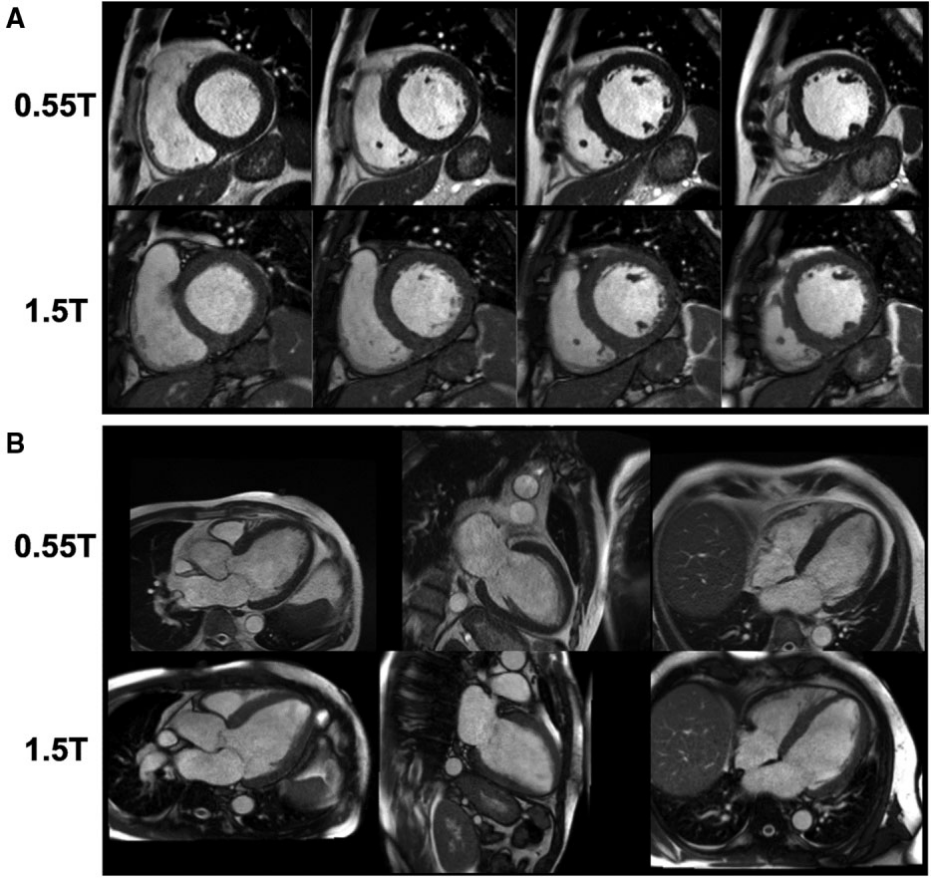
Image comparisons of breath held cine bSSFP at 0.55 T and 1.5 T, taken in the short axis (*A*) and the long axis (*B*). These images were taken from a patient with non-ischaemic cardiomyopathy (Original Image from Ref.^31^).

A 0.35 T MRI-radiotherapy system with superconducting magnet and high-performance gradients has also been demonstrated for CMR. Simonetti and Ahmad^33^ used compressed sensing to generate high-quality CMR images with this 0.35 T system configuration. As described in *Figure 6*, sufficient image quality was maintained, even when SNR was degraded to levels at ∼0.35 T. The strategy of acquiring images on LF systems using high flip angles has been shown to have diagnostic potential. Rashid *et al*. compared CINE imaging CMR-on 6–7 healthy volunteers on both the 0.35 T and a 1.5 T system using a range of flip angles governing blood myocardium contrast. Leveraging lower SAR restrictions at 0.35 T, they found blood-myocardium CNR was boosted at flip angles over 90°, with the optimum CNR achieved at 130°. Subjective image quality between 0.35 T images achieved at flip angles of 110° and 130° compared to 1.5 T images acquired at 90° was identical.^27^ Varghese *et al.* compared the feasibility of assessing cardiac function and flow using CINE and phase-contrast CMR respectively at 0.35 T, 1.5 T, and 3 T on six healthy volunteers, utilizing a high flip angle of 110° for 0.35 T images as per Rashid *et al*.’s findings. Good diagnostic image quality was achieved at 0.35 T for all scans, although blood-myocardium CNR was significantly lower at 0.35 T compared to 1.5 and 3 T. However, quantitative cine and flow measurements between 0.35 and 1.5 T did not differ significantly.^28^

It must be noted that the sample sizes of these studies were relatively small, and some were conducted on healthy volunteers. Whilst SNR has been used as a standard metric for comparison, clinical utility (correct diagnosis in reasonable imaging time) is paramount for a routine deployment. In addition, the above validation studies were conducted on superconducting systems using high-performance gradients. Nevertheless, the preliminary results of these studies are promising, and with continued research and technological advancements, they may be a viable solution in LMICs in the future.

### Opportunities for interventional CMR

Interventional CMR (iCMR) holds great promise in improving the accuracy and safety of invasive cardiac procedures. CMR enhances visualization of cardiac anatomy without radiation or iodinated contrast.^125^^,^^126^ The bSSFP sequence also allows for real-time intra- and post-procedural monitoring. There have been increasing numbers of successful pre-clinical human studies in CMR-guided right heart catheterization^113^^,^^126–131^ and ablation of atrial flutter,^132^^,^^133^ clinical translation of iCMR remains a challenge. iCMR devices (guidewires, catheters, etc.) are made from paramagnetic materials such as nitinol or stainless steel, which are susceptible to RF-heating increasing quadratically with field strength.^134^^,^^135^ LF CMR may mitigate the risk of thermal injury as reported in the successful right heart catheterization of 7 individuals using a 0.55 T superconducting scanner where 9 of 16 catheters evaluated were free from heating.^113^

Moreover, in a swine model, artefacts from high-susceptibility materials like stainless steel were indistinguishable across 0.55 T, 1.5 T, and 3 T, meaning device visibility is maintained at LFs with reduced heating for some metals.^114^ LF CMR pre-procedural planning and post-procedural assessment are demonstrated in a swine model when Kolandaivelu *et al.*^136^ used LF (0.55 T) native T1-weighted contrast CMR to assess and characterize tissue necrosis from cardiac ablation, especially with acetic acid chemoablation. This work indicates the potential of using LF CMR for accurately delineating myocardial lesions and necrosis to guide cardiac ablation.

Though still in its inception, LF iCMR is undoubtedly worthy of more significant investment and research in HICs and LMICs. Though its cost is yet to be determined, the advantage of minimally-invasive procedures without any risk of radiation is substantial, especially for children and pregnant women. Arguably, LF iCMR can afford even greater benefits for LMICs due to high perioperative mortality rates associated with surgical and general anaesthetic complications.^137^

## What would be the cost advantages of LF CMR?

The expense of an MRI unit consists of purchase, siting, maintenance, and operational costs. Purchase costs are determined by magnet type, magnetic field strength, gradient strength, RF coil type, bore diameter, installation cost, and warranty.^77^ There is limited published data available on the cost of MRI, as most of the information is proprietary. In general, the average purchase cost of a new commercial MRI machine is estimated at up to €1 million (1.2 million USD) per tesla. Factoring in installation costs, maintenance of the MRI suite, built-in safety measures, and patient support areas, the total capital cost needed to procure and site a single unit can reach €3–5 million (3.6–6 million USD).^138^ Indeed, a 2010 Belgian study of 28 hospitals found that the average sales price of a standard-configuration whole-body MR unit installed in 2006–08 exceeded €1 million for 1.5 T and €1.5 million for 3 T scanners (1.2–1.8 million USD). Incorporating organ-specific and imaging technique-specific software and higher gradient strength hardware would require several hundred thousand euros in addition.

Furthermore, one-off building adjustment costs varied from €160 000 to €240 000 for 1.5 T and €230 000 to €330 000 for 3 T units.^139^ A recent descriptive study found the total installation cost of a 1.5 T scanner in a tertiary hospital in India was just over 1 million USD.^140^ CMR is more expensive, with a typical 1.5 T scanner costing between €1.6 and 2 million ($2–2.5 million), including purchasing and siting adaptations.^33^ LF CMR should theoretically be less expensive by having reduced magnetic field strength. However, available contemporary LF CMR systems used in research employ superconducting magnets and high-performance gradient systems, which invariably keep costs high. There is currently a push to make the commercial versions of these systems affordable and more accessible.

The value of MRI hinges on clinical relevance, whereby improving patient outcomes and satisfaction is balanced against lowering costs.^141^ HICs studies have demonstrated the cost-effectiveness of CMR with improvements in quality-adjusted life years (QALYs), predominantly for evaluating suspected CAD, which carries the most significant disease burden in HICs.^142–146^ In the case of LF CMR, it can be argued that the longer scan duration can be offset by the lower unit acquisition price and the costs of installation and maintenance. The main stakeholders’ interests, i.e. the patient, the referring physician, the hospital/healthcare system, and the payer, must all be balanced. Producing comfortable, accurate, efficient, and rapid scans with high-quality reports are crucial to lowering the cost for the healthcare system and the individual. Furthermore, MRI value varies globally and must be tailored to local healthcare needs, healthcare systems, human resource capacity, and infrastructure.

## The benefits of sustainable CMR worldwide

### Rapid CMR

Simplifying MRI hardware and software is an attractive solution to reduce its cost and complexity. Only a few key organ-specific imaging techniques may be needed in many protocols to generate most data required for diagnosis.^141^ The TIC-TOC study used an abbreviated non-contrast CMR protocol on a 1.5 T scanner to assess cardiac iron overload in thalassaemia patients in Thailand. Overall, 123 scans took place over two 12-h days with a mean scan duration of only 8.3 ± 2 min.^76^ Similar findings were reported in a previous multi-centre Brazilian study reporting median scan times of 5.2 min.^147^ Though these patient populations were highly specific, these proof-of-principle studies demonstrated the feasibility of using ultrafast CMR scanning in assessing a burdensome health problem in LMICs.

Similarly, Menacho *et al*. developed a short CMR protocol of 15 min duration to evaluate LV function, volumes, and scarring. Following training, this protocol was implemented in 100 referred patients in Lima, Peru, with an average scan time of 18 ± 7 min. Scan results were demonstrated to change subsequent management in 56% of participants in the following year.^148^ Notably, the sustainability of this training programme has been validated by its continuous implementation in six centres in Peru and its adoption in centres across Argentina, South Africa, and India.^36^ Rapid CMR may also benefit HICs in the long term by reducing scanning times, improving throughput, and enhancing access for deprived populations and rural communities.

### Encouraging local production

Encouraging local manufacturing is a crucial strategy for facilitating sustainable diagnostic imaging.^149^ For example, the Government of India’s initiative (coordinated by SAMEER) on increasing MRI affordability focuses on local production of a 1.5 T superconducting magnet using cryocooling technology and indigenously developing all constituent hardware and software components.^150^ Similarly, private players in India, such as Voxelgrid Innovations, in partnership with Tata Trust, have developed whole body 1.5 T scanners using new helium gas technology, designed to conserve power and reportedly scans four times faster than other commercially available scanners.^151^^,^^152^ With heavy government-backed policy, China is another dominant player in local manufacturing of diagnostic devices such as the Brivo MRI^153^ and, more recently, an ultrawide bore MRI machine debuted. More public–private partnership is required within countries and across borders to encourage local LMICs MRI production.

### Investing in training, research, and global collaboration

In the open-access open resource imaging era, making publicly available medical technology components, including the blueprints and code, allows engineers and physicists from different locations to develop products tailored to the local environment and specific needs. This fosters research and innovation, enabling local manufacturing to substantially lower maintenance costs and offset the micro- and macro-economic divide.^154^ The imaging workforce encompasses many skilled stakeholders; their scarcity forms a key barrier to implementing sustainable radiological services.^155^ Access to medical education is also a challenge in rural regions. The training of technicians, medical physicists, and engineers is also vital. Building collaborative links between HIC and LMICs are essential to filling the gap in global radiology education. Notably, the international non-profit organization RAD-AID has supported radiology education and imaging services across many LMICs and rural regions of HICs.^156^^,^^157^

## Future prospects—rich opportunities for clinical integration

### How can LF CMR benefit cardiac imaging in HICs

The increasing flow of knowledge from HF CMR technologies and applications is advancing the capabilities of CMR at lower field strengths. This move should be handled with care as it is not simply a matter of copying practices and protocols from a higher field to a lower one. One area that requires further attention concerns the development of LF CMR technology to improve healthcare in rural/deprived regions of HICs where access to diagnostic imaging is limited. Knowledge gained from experience with LF CMR in LMICs can likely be re-applied to its implementation and application in HICs. LF MR magnets can improve access to care in HICs, having the added benefit of being lightweight and having a smaller magnetic footprint while providing similar diagnostic performance as HF MR systems. One example of a critical clinical application would be the use of CMR in neonates and infants for diagnostic imaging of congenital heart disease.^158^^,^^159^ Here, dedicated small size, LF MR systems can bring imaging advances to paediatric and neonatal intensive care units where the youngest and most vulnerable patients deserve the best medical care and treatment. MRI in patients with tetralogy of Fallot, aortic arch anomalies, and Fontan circulation does not require high fidelity spatial resolution offered by expensive HF MRI scanners but can be appropriately performed with the performance provided by LF CMR.^160^ Likewise, LF CMR might be clinically meaningful for the early identification of shunting in patients with patent foramen ovale.

One other potential clinical application of mobile LF CMR could include the assessment of myocardial iron overload in thalassaemia major, which is a significant prognosticator of myocardial injury.^161^ The MRI-derived effective relaxation time T_2_*—the MRI surrogate for myocardial iron concentration—is very much prolonged at lower magnetic field strengths. This advantage will likely be beneficial for enhancing diagnostic image quality in MRI-based iron level assessments of the heart to guide thalassaemia major treatment.^162^ Here, knowledge and experience of rapid CMR protocols implemented in LMICs could transfer to HICs.

### Bringing MRI to where people need it

A recent single-centre study has accelerated the development of portable MRI, demonstrating the feasibility of using the first commercially available point-of-care 0.064 T MRI in neurocritical care.^163^ Portable LF MRI may reduce cost and decentralize imaging services, enabling access to rural and remote communities in LMICs and HICs. For example, in developed countries, mobile LF MRI implemented in ambulance cars or small vehicles, state-of-the-art healthcare can be brought to almost any facility’s doorstep, providing easy access to out-of-hospital medical care to patients. Furthermore, its usage in areas including the emergency departments and intensive care units where ferromagnetic materials may be nearby may well be permitted.^34^ Portable, low-cost MRI utilizing Halbach arrays and pre-polarized MRI technology has been studied extensively in the brain and extremity imaging.^164^ These may find use in CMR, paving the way for a viable future where point-of-care CMR could replace echocardiography as the new workhorse of cardiac imaging.

### AI-enabled CMR

Deep learning (DL) is increasingly applied to cardiovascular imaging to enhance image resolution, acquisition, speed, and reconstruction.^165^^,^^166^ A recent study on whole heart CMR demonstrated that reconstructed images from the trained neural network have significantly improved image quality and reduced artefacts whilst shortening acquisition time.^167^ DL has also shown promising results when applied to LF MRI in recovering image quality.^82^ Integrating DL in LF CMR may boost SNR, reduce acquisition time and streamline LF-CMR examinations, including automated planning of scan planes and cardiac views and AI-guided reading and classification of findings. These efforts will all help to make MRI universally available in HICs and LMICs. Indeed, DL technology is being rapidly incorporated into all aspects of imaging workflow and delivery in HIC, with ongoing questions over whether AI is set to replace human radiologists in the near future.^168^ Whilst there are numerous barriers to AI adoption in LMICs, it can potentially yield remarkable benefits in resource-poor communities. For instance, could AI-guided image interpretation overcome the workload burden in large populations with few radiologists whilst improving quality assurance and safety?^156^

## Conclusion

The increasing interest and enthusiasm for developing low cost, sustainable, yet high-quality LF MRI technologies hold great promise for improving global access to essential diagnostic imaging. This opportunity should serve as a catalyst to incentivize relevant stakeholders to invest in further research and development to create sustainable healthcare ecosystems to propagate this technology. This may help level the playing field in cardiology and other disciplines across different socio-economic divides in LMICs and HICs.

**Conflict of interest:** C. Q., S. M., E. L., V. S., D. H., J. O., J. B., R. L., H. D., U. C. A., N. J., N. A.B. N., and R. M. all declare that they have no conflict of interest. T. A. has received support from the NIHR Applied Research Collaboration ARC Wessex and Health Education England South East and was funded by an NIHR ARC Wessex and Health Education England South East Researcher Enhancement Award grant. O. S. has a grant from Siemens which goes towards the funding of the research conducted at The Ohio State University. They are also an inventor on a US patent related to low field cardiac MRI. A. C-W. is an investigator on a US Government Cooperative Research and Development Agreement (CRADA) with Siemens Healthcare, and Siemens participated in the modification of the NHILBI MRI system from 1.5T to 0.55T under this CRADA. T. N. is the founder, CEO and shareholder of MRI.TOOLS GmbH, Berlin, Germany. S. A. is an Academic Clinical Fellow funded by the United Kingdom National Institute for Health Research (NIHR). The views expressed in this publication are those of the authors and not necessarily those of the NHS, NIHR, NIH and other author-affiliated institutions.
